# Can the Weight of an External Breast Prosthesis Influence Trunk Biomechanics during Functional Movement in Postmastectomy Women?

**DOI:** 10.1155/2017/9867694

**Published:** 2017-09-24

**Authors:** Katarzyna Hojan, Faustyna Manikowska

**Affiliations:** ^1^Department of Rehabilitation, Greater Poland Cancer Centre, Poznan, Poland; ^2^Department of Pediatrics Orthopedics and Traumatology, Poznan University of Medical Sciences, Poznan, Poland; ^3^Motion Analysis Laboratory, Poznan University of Medical Sciences, Poznan, Poland

## Abstract

**Introduction:**

Recent papers indicate that one-side mastectomy can produce deleterious effects on the posture and musculoskeletal system. This study was conducted to better understand the underlying mechanisms involved in trunk motion in external prosthesis users.

**Objective:**

The aim was to evaluate the changes in surface electromyographic (SEMG) activity of the erector spinae muscles (ES) in postmastectomy women with and without breast prostheses during functional body movement tests.

**Methods:**

In 51 one-side postmastectomy women the SEMG muscle activity of bilateral ES was measured during symmetrical and asymmetrical dynamic activities in a counterbalanced manner with different weights of the breast prosthesis. Range-of-motion measurements were taken for forward bending, backward bending, lateral bending, and rotation.

**Results:**

The mean level of the ES activity in the lumbar region was not affected by the weight of the external breast prosthesis during most of the functional body tests (*P* > 0.05). The activity of ES during functional body tests with and without different external breast prostheses did not differ between the two sides of the trunk (mastectomy and nonmastectomy) for most of the movement tests (*P* > 0.05).

**Conclusion:**

The lumbar ES activity during functional tests is not associated with the weight of the external breast prosthesis in postmastectomy women.

## 1. Introduction

Recently, it has been estimated that about 90% of women undergoing mastectomy use a breast prosthesis permanently or during the waiting time preceding breast reconstruction [1]. The standard full external breast prosthesis, which is molded to the natural shape and weight of the woman's breast and worn in a special bra, should be used as a first choice after total mastectomy. Even though more than 50% of women wear full weight breast prosthesis, the other use a lighter type of prosthesis or even home-made prosthesis of cotton, rice, and so on [2]. This group of women is dissatisfied with various aspects of external prostheses and report dissatisfaction with incorrect fit, restrictive choice of clothing, and difficulty in dressing, discomfort, and prostheses' weight or cost [3–7]. Even though they claim that the method of breast restoration has no apparent impact on their positive attitude to themselves and their life [8], almost half of women with external breast prosthesis say that the prosthesis limits them in doing sports [2, 8].

The studies indicate that intense and frequent physical activity is related to better functioning in daily life and reduction of fatigue pain and depressive symptoms in breast cancer patients [8, 9]. Postmastectomy women complain about the decrease in functional and psychological status, disability, and the postmastectomy syndrome [10]. Recent clinical studies have underlined the functional state as the basis for rehabilitation diagnosis and treatment in breast cancer patients [11, 12].

In our study, we wanted to assess whether “the golden standard” of using external breast prosthesis with the weight and size of the amputee's breast is reasonable. Matory et al. [13] investigated women after partial mastectomy, with breast asymmetry. The study demonstrated that those women were satisfied and did not complain about breast asymmetry [13]. This satisfaction is not necessary based on objective criteria but is the result of retaining the original breast. Moreover, previous studies [2, 7] showed that almost half of women after mastectomy chose a lighter prosthesis, whose weight did not equal that of the other breast, and used it as their first choice. We are not aware of any study on the association between the weight of the external breast prosthesis and body movements in postmastectomy women.

Therefore, the aim of the study was to determine if the weight of a breast prosthesis can affect the biomechanics of the trunk during functional movement. In particular we aimed to

1. determine if the level of activity of the erector spinae muscle (ES) during trunk movement is affected by the weight of the external breast prosthesis;
2. establish the effect of weight of the external breast prosthesis in women after one-side mastectomy on the bilateral symmetry of activity of ES during trunk symmetrical movement;
3. determine if the anthropometrical features and time passed since mastectomy affected the level of activity of ES during trunk movement with different external breast prostheses.

To achieve the aim of the study we measured the EMG activity of ES and compared the level of muscle activation: in different types of breast prostheses and between sides of the body during the EMG test.

## 2. Material and Methods

### 2.1. Design

This was a single center, observational, clinical study. Patients were recruited via poster invitation in the local cancer center according to study criteria between June 2015 and January 2016. The study was conducted in the Department of Rehabilitation and it was approved by the local ethics committee at Poznan University of Medical Sciences. The study was conducted in accordance with the Declaration of Helsinki. All the participants were informed in detail about the research protocol and gave their informed consent to participate in the study.

### 2.2. Participants

Fifty-one patients with a history of unilateral mastectomy due to breast cancer, aged 35–70 years, were divided into two subgroups: group R (surgery performed on the right side) and group L (surgery performed on the left side). Inclusion criteria for study participation is comprised of being female, having a history of unilateral breast surgery (modified radical mastectomy), and being in otherwise good general health (Grade 0 or 1 of “Eastern Cooperative Oncology Group Performance Status”). Exclusion criteria for study participation included quadrantectomies, lymphoedema, peripheral nerve damage, neurologic diseases, cognition deficits, history of significant orthopedic problems (including bone metastases), breast reconstruction, radiotherapy, engagement in activities that could originate posture asymmetries or any type of treatment for posture correction, and musculoskeletal (especially low back pain, myopathy, and fatigue) or cardiovascular impairment or disease (except breast cancer) that affected body motion patterns or postural control.

### 2.3. Measurements

Information regarding patients' age and anthropometric measures including body mass, height, weight of operated breast, and time after surgery was also collected.

Measurements were performed in each postmastectomy woman with four different wear-types of breast prostheses: A, not wearing a prosthesis; B, wearing a prosthesis weighing 10 grams; C, wearing a prosthesis weighing 50% of the total breast mass subtracted during the mastectomy; and D, wearing a prosthesis of equal weight to the operated breast (determined intraoperatively during the mastectomy).

The selected surface EMG (SEMG) activity of ES during the posture tests for each prosthesis was calculated using 5 signal repetitions. SEMG data were collected using a 4-channel EMG device (Noraxon TeleMyo 400, Noraxon, Scottsdale, AZ, US). EMG signals were detected using pregelled Ag–AgCl (BIO LEADLOK) electrode pairs applied at the L3-4 level over ES muscles (about 4 cm lateral from the midline of the body). The center-to-center electrode distance was 2.5 cm, and electrodes were longitudinally oriented along the muscle fibers. A reference electrode was taped onto the patient's left wrist. EMG signals were recorded according to SEMG for the Noninvasive Assessment of Muscles (SENIAM) guidelines [14, 15]. Those data were analyzed using MyoResearch Master Edition 1.06 XP software.

*Testing Protocol*. The patients were measured with and without different types of prostheses in the following phases:

- Bending of the torso/returning to the neutral position: the woman tested performs a full torso bend forward (5 sec); next she remains in a position of maximum bending loosely hanging (bending-relaxing) for 5 seconds and returns to a neutral position (5 sec).
- Straightening up/returning to the neutral position: the woman tested stands in a comfortable and natural position for 5 seconds; next she bends backward (5 sec) and returns to a neutral position (5 sec).
- Rotation of the torso to the right and left: the woman tested performs a rotation of the torso to the right and back to the neutral position (7-8 sec).
- Rotation of the torso to the left: after that, the patient tested performs a rotation of the torso to the left and back to the neutral position (during the next 7-8 sec).
- Side-bending of the torso to the right: the woman tested performs bending of the torso to the right and back to the neutral position (7-8 sec).
- Side-bending of the torso to the left: the woman tested performs side-bending of the torso to the left and back to the neutral position (7-8 sec).

The mean value of the SEMG (mV) signal: the work of the muscle to the right and left was analyzed during each of the moves carried out in the various phases of the test. To prevent muscle fatigue, there was a 5-second rest period between the motions. The values obtained for the various phases of the test were compared between the measurements conducted without prosthesis, next with the following prostheses: B, C, and D. The mean value of the SEMG signal was analyzed for the following test phases:

1. Trunk flexion: bending forward movement (eccentric work of the spinal extensor) and return movement to the starting position (concentric work of the spinal extensor).
2. Sagittal extension: extension backward in the sagittal plane.
3. Extension/flexion ratio: the value of the extension/flexion ratio measured during the II phase of the test.
4. Rotation right: rotation to the right in the transverse plane (comparison of muscle activity on the right and left side).
5. Rotation left: rotation to the left in the transverse plane (comparison of muscle activity on the right and left side).
6. Lateral flexion right: bending to the right in the frontal plane (comparison of muscle activity on the right and left side).
7. Lateral flexion left: bending to the left in the frontal plane (comparison of muscle activity on the right and left side).

### 2.4. Data Collection

The EMG signals were bandpass filtered between 20 Hz and 500 Hz and then digitized at a sampling rate of 1000 Hz. The digital signals were then full-wave rectified and filtered using Lancosh FIR digital filters. The activity onset time of each muscle was defined as the point at which the signal amplitude exceeded the mean amplitude plus three standard deviations (SD) during the 200 ms before the start of the STS procedure [14, 16]. We performed visual inspection of each trial to determine whether the EMG value and graph were repeatable for each subject. If there were significant changes of the value or the graph pattern, we rejected the data and repeated the data collecting process.

The degree of muscle activity was assessed for each muscle by calculating the root mean square (RMS) moving window of 300 ms duration of EMG data. The EMG activity was normalized against the mean RMS of a reference voluntary contraction (RVC) for each muscle [16] and was calculated for the period during the entire STS task [17]. The protocol was created by our programmer, who developed a custom-written algorithm. We entered the data in this automatic code and received a normalized result.

### 2.5. Outcome Measure

The outcome measure of the study was the difference in the activity of ES, that is, the difference in the level of muscle activity with different weights of external breast prosthesis during functional tests.

### 2.6. Data Analysis

To determine whether the weight of the external breast prosthesis contributed to any posture changes and whether the weight of the prosthesis should be altered depending on the duration of time since mastectomy, statistical analyses were performed based on paired *t*-tests and multivariate ordinary least square (OLS) regression. For each wear-type, different posture outcome measures for the operated and nontreated side of the same patient were compared using paired *t*-tests. To adjust for other potential confounders, the following factors were controlled for in the OLS model: BMI (kg/m^2^), time from operation (months), and age (years). Patient characteristics were compared between patients from groups R and L. All data analyses were completed with STATA (STATA Corp., TX, USA).

## 3. Results

### 3.1. Participants Characteristics

Fifty-one patients with a history of mastectomy were included in the study. Twenty-nine of these patients had undergone a mastectomy on the right side (group R) while 22 underwent one on the left side (group L). The overall mean (SD) age was 58 (11.4) years. In group R, the mean (SD) age was 58 (12.4) years. In group L, the mean (SD) age was 58 (9.9) years. The average height (cm) and weight (kg) were 163.7 and 74.2 respectively, with the average BMI equal to 27.7 kg/m^2^. These differences were not statistically significant, except that the women in group L were slightly taller and heavier than those in group R, but this did not result in a difference in BMI between the groups (Table 1).

### 3.2. Differences between ES Activity with Many Types of External Breast Prostheses in Functional Position Tests

We compared both sides of the trunk (R and L). The mean level of activity of ES was not affected by the weight of the external breast prosthesis during all the functional body tests (*P* > 0.05). There was no change in the activity of ES between the two sides (the mastectomy and nonmastectomy side of the body) in most symmetrical movements of the trunk (*P* > 0.05) even though different weights of external bra were used.  Table 2 presents the means of ES activity with prostheses of different weights and without prosthesis during position tests.

### 3.3. Correlation

BMI, time after mastectomy, or age in the women studied did not influence the mean of ES activity during the dynamic EMG tests with different weights of the external breast prosthesis (*P* > 0.05) or without prosthesis. Only the time after mastectomy variable positively correlated with 10 g prosthesis in the flexion test (Table 3).

## 4. Discussion

Good quality and appropriated fitting of the external breast prosthesis are crucial for women after mastectomy [2–4, 6, 7]. However, research in this area is still very limited. It is still unclear whether the weight of the breast prosthesis affects the physical activity and daily life activities of women after mastectomy [6, 7, 18]. The first aim of our study was to determine whether the weight of the external breast prosthesis affected the level of muscle activity. We found that the differences in the mean level of ES activity during trunk movement in different types of external breast prostheses were not statistically significant in most of the positions. Moreover, the weight of the external prosthesis did not affect the symmetry of the activation level of ES between the two sides of the body. The mean levels of muscle activation differed between the operated and nonoperated side with different prostheses. Only single tests with different prostheses demonstrated statistically significant results but they were not clinically useful. Furthermore, there was no influence of BMI and age on ES activity regardless of the weight of the prosthesis. Similarly, time elapsed since the surgery did not affect the level of muscle activity during the dynamic muscle test without prosthesis and with different weights of the prosthesis.

Our research shows that the weight of the prosthesis is not an important factor affecting the postmastectomy women's posture during physical activity. Differences of ES muscle activity on both sides of the body during trunk movement with different weights of the prostheses were not clinically important. It could indicate that total weight compensation of the amputee's breast is not necessary for proper biomechanics of the trunk. It has been shown that the movement of the breast in healthy women during physical activities is excessive because of the lack of internal breast support. To reduce breast motion, external breast support, that is, a sports bra, is used [2]. In our study, women wore a special bra with space for breast prosthesis. We changed only the weight of the prosthesis, but the support of the breast was the same. We did not notice any significant differences in muscle activity during trunk movement in numerous positions in women wearing different types of external prosthesis. This study's results showed that the level of ES does not depend on the weight of the external breast prosthesis.

We assessed erector spinae EMG activity during different daily practical trunk movements using standardized assessment protocol of tasks, and we did not find significant differences in the level of muscle activity between the sides of the body with different types of the prosthesis used. We used SEMG because the method can provide useful information about muscle's functional status, and if properly applied it gives reliable results [19, 20]. SEMG is widely used to analyze back muscle activity and many research studies were conducted to understand the SEMG techniques and their application to the analysis of low back muscles for classifying healthy subjects and low back pain patients, trained and nontrained subjects, and subjects under rehabilitation treatments as well as access the muscle's activity during labor tasks, sports practice, or daily life activities [14–16, 20–22]. We measured ES because it is lateral to the multifidus muscle and forms the prominent dorsolateral contour of the back muscles in the lumbar region.* In vivo* ES techniques have been employed to measure spine kinematics during various physical activities [23, 24]. The ES in the region examined is the back muscles, which are active the whole day through; therefore they are of particular relevance [25, 26].

Very limited data on body or trunk muscle activity in postmastectomy women are available. Only studies on posture after mastectomy were conducted [27–30] but these did not include dynamic measurement. In Hooper et al. trial [31], the authors presented evidence on the mechanical consequences of reduction mammoplasty on the low back. They demonstrated that the breast size influenced moments and loads acting on the lumbar spine during the isometric and dynamic tasks. Due to reduced breast mass, women after reduction mammaplasty were able to lift faster with a decreased moment during isometric tasks. Those authors observed women in early period (to 2 months) after mammoplasty and in frontal movements. In our previous study [32], we observed an important influence of the external breast prosthesis on gait parameters in postmastectomy women (especially in a young age).

In the present study, we did not observe any correlation between ES muscle activity and age, time after mastectomy, or anthropometrical parameters. Moreover, lumbar spine kinematics have not been reported in postmastectomy women wearing an external breast prosthesis. Another of our studies [27] showed that the trunk muscles of the affected side (during arm movement) presented significantly less activity, than those on the nonaffected side of the body even if these muscles were not in the field of the surgery or radiotherapy. This can result in pain, muscle imbalance, and movement disorders. Even though we observed a significant increase in EMG activity of the ES on the nonoperated side, the weight of an external breast prosthesis did not contribute to posture changes in postmastectomy women. Other researchers showed that only external load of at least 15% of body weight can cause postural changes of the trunk [33, 34].

Women after mastectomy, who did not take part in rehabilitation, had on average a 40% strength deficiency compared to the control group and achieved only 57% of the healthy women's capability for extensor muscles and 49% for flexor muscles of the spine [35].

None of the previous studies considered that the weight of the external breast might not affect trunk muscle activity. This paper is one of the first objective feasibility studies on trunk analysis with the use of different weights of external breast prosthesis in women after mastectomy in different body positions (similar to those assumed during regular daily activity). Our results provided experimental evidence for the above-stated suggestions. These findings should become a part of integral diagnosis for the development of best standards of practice in the fitting and supply of these prostheses in postmastectomy women.

Weakness of this study was related to its pilot character, that is, it only investigated the relationship between muscle activity and the weight of the external breast prosthesis. Since it was a pilot study, the number of participants was limited.

## 5. Conclusions

From a clinical perspective, the lumbar ES activity is not associated with different weights of the external breast prosthesis in postmastectomy patients during functional tests. Our results provide preliminary evidence that the weight of an external breast prosthesis in postmastectomy women is probably not an important factor for kinematics of the trunk. This study needs to be continued in a large group of patients, using other types of multidimensional biomechanics equipment.

## Figures and Tables

**Table 1:** Baseline characteristics of the participants.

Parameters	All study participants		Operation side, right (R)		Operation side, left (L)		
	(*N* = 51)		(*N* = 29)		(*N* = 22)		
Variables	Mean	SD	Mean	SD	Mean	SD	*P* value

Age (years)	58.18	11.39	58.10	12.43	58.27	9.91	0.917
Height (cm)	163.75	5.6	163.07	6.15	164.64	4.66	**0.048**
Weight (kg)	74.22	11.23	72.79	11.32	76.09	10.89	**0.038**
BMI (kg/m^2^)	27.73	4.29	27.47	4.55	28.08	3.93	0.314
Time after mastectomy (month)	30.63	41.78	29.59	41.48	32.00	42.37	0.684

Breast size	Number of women	% of all	Number of women	% of R	Number of women	% of L	

A	0	0	0	0	0	0	
B	9	17.5	5	17	4	18	
C	29	57	16	55.5	13	59	
D	13	25.5	8	27.5	5	23	
E	0	0	0	0	0	0	

SD: standard deviation; BMI: body mass index.

**Table 2:** Comparison between the right (R) and left (L) side mastectomy regarding the mean (SD) EMG activity of erector spine muscles during different phases (position tasks) for different types of breast prosthesis or no bra.

Position test	Type prosthesis	Operated side: R	Nonoperated side: L	*P* value	Operated side: L	Nonoperated side: R	*P* value
Flex. relax. post. mean	A	51.24 (31.66)	60.6 (39.5)	**0.039**	50.02 (25.06)	51.54 (26.52)	0.647
	B	49.15 (32.35)	57.56 (42.92)	**0.045**	52.2 (24.36)	54.45 (28.36)	0.555
	C	50.75 (31.97)	59.49 (44.05)	**0.05**	52.01 (25.44)	48.44 (24.72)	0.16
	D	50.74 (33.61)	63.38 (47.31)	**0.013**	54.34 (25.04)	55.23 (30.63)	0.812
	*P* value	0.997	0.976		0.954	0.854	

Ext. post. mean	A	15.28 (15.58)	14.9 (13.14)	0.74	11.01 (5.9)	11.68 (6.27)	0.223
	B	11.38 (4.3)	12.12 (6.95)	0.568	11.15 (6)	12.32 (4.33)	0.167
	C	11.9 (4.79)	11.53 (6.59)	0.708	10.57 (7.66)	11.89 (4.82)	0.19
	D	12.17 (6.52)	12.78 (8.22)	0.53	13.51 (11.09)	12.65 (4.84)	0.726
	*P* value	0.47	0.622		0.618	0.925	

Trunk flex./ret. mean	A	42.98 (21.9)	45.53 (25.31)	0.417	42.57 (17.87)	42.68 (21.67)	0.961
	B	41.65 (24.26)	47.8 (29.18)	0.06	46.17 (19.6)	44.99 (22.35)	0.665
	C	39.75 (22.23)	46.36 (28.2)	0.066	44.9 (19.37)	42.82 (18.66)	0.484
	D	42.07 (23.06)	48.38 (30)	0.082	48.58 (19.18)	49.32 (26.19)	0.812
	*P* value	0.97	0.986		0.766	0.744	

Ext.-flex. ratio	A	3.52 (10.21)	3.09 (7.56)	**0.443**	1.55 (0.69)	1.48 (0.53)	0.278
	B	3.98 (12.28)	3.13 (8.35)	**0.322**	1.33 (0.56)	1.4 (0.64)	0.253
	C	4.05 (12.79)	3.51 (10.1)	**0.353**	1.4 (0.48)	1.42 (0.56)	0.813
	D	4.51 (15.02)	3.19 (8.77)	**0.337**	1.35 (0.55)	1.29 (0.52)	0.356
	*P* value	0.995	0.998		0.579	0.739	

Trunk flex./ret. mean	A	57.84 (27.07)	67.45 (37.11)	**0.041**	61.92 (26.59)	59.53 (29.14)	0.356
	B	55.3 (27.09)	63.88 (37.45)	0.09	57.09 (25.15)	57.34 (27.72)	0.927
	C	53.64 (22.45)	61.43 (31.87)	0.083	58.31 (23.56)	57.73 (29.73)	0.855
	D	51.39 (23.17)	57.81 (27.01)	0.147	60.95 (24.77)	59.39 (29.48)	0.498
	*P* value	0.85	0.805		0.913	0.992	

Rotation right mean	A	23.5 (12.96)	29.09 (18.07)	0.123	27.82 (11.64)	22.18 (9.48)	**<0.001**
	B	21.09 (8.23)	27.68 (17.88)	**0.035**	30.15 (11.96)	24.79 (11.21)	**0.006**
	C	22.08 (8.75)	28.85 (17.24)	**0.023**	29.23 (11.37)	24.47 (9.93)	**0.008**
	D	23.11 (10.93)	28.3 (17.41)	0.092	28.68 (10.22)	25.2 (9.69)	**0.03**
	*P* value	0.867	0.994		0.92	0.758	

Rotation left mean	A	25.84 (17.14)	25.55 (16.81)	0.94	23.82 (10.13)	26.56 (12.18)	0.159
	B	22.78 (11.32)	26.56 (17.04)	0.224	26.34 (9.61)	27.09 (9.15)	0.667
	C	23.02 (10.25)	25.31 (17.5)	0.469	23.51 (10.24)	28.16 (12.11)	**0.029**
	D	24.64 (11.07)	27.37 (19.16)	0.44	23.49 (8.7)	28.45 (11.19)	**0.024**
	*P* value	0.836	0.979		0.725	0.938	

Lat. flex. left mean	A	18.87 (15.3)	19.48 (13.87)	0.83	16.85 (7.83)	19.81 (7.18)	**0.082**
	B	15.62 (7.92)	19.14 (13.07)	0.128	17.88 (6.61)	20.35 (8.13)	**0.033**
	C	16.4 (8.46)	18.87 (13.19)	0.248	18.96 (8.49)	20.54 (8.69)	0.233
	D	17.47 (9.93)	20.09 (16.91)	0.327	19.42 (7.51)	22.65 (8.77)	**0.015**
	*P* value	0.768	0.993		0.688	0.679	

Lat. flex. right mean	A	18.62 (13.72)	20.66 (17.05)	0.512	20.85 (9.48)	18.33 (6.68)	**0.036**
	B	16.76 (7.39)	21.82 (16.28)	0.058	21.47 (9.81)	19.17 (7.54)	**0.051**
	C	17.8 (9.04)	20.78 (14.6)	0.168	20.28 (9.47)	19.84 (9.28)	0.639
	D	18.08 (9.14)	21.3 (17.28)	0.267	20.1 (8.52)	21.94 (8.81)	0.182
	*P* value	0.941	0.995		0.962	0.501	

**Table 3:** Correlation between different position tests and BMI, time after mastectomy, and age.

Position test		BMI		Time after mastectomy		Age	
	Type prosthesis	*r*	*P* value	*r*	*P* value	*r*	*P* value
Flex. post. mean	A	0.071	0.643	0.251	0.096	0.143	0.35
	B	−0.074	0.635	0.399	**0.007**	0.059	0.703
	C	0.076	0.63	0.221	0.155	0.174	0.266
	D	0.116	0.454	0.252	0.1	0.062	0.687

Ext. post. mean	A	−0.292	0.052	0.163	0.286	0.005	0.973
	B	−0.131	0.396	0.1	0.518	0.079	0.61
	C	−0.005	0.973	0.116	0.459	0.17	0.276
	D	−0.001	0.994	0.139	0.37	−0.054	0.727

Trunk flex./ret. mean	A	−0.128	0.402	0.196	0.197	0.066	0.665
	B	−0.038	0.806	0.237	0.122	0.066	0.668
	C	−0.069	0.658	0.151	0.333	−0.055	0.727
	D	−0.068	0.662	0.276	0.07	0.039	0.8

Left ext.-flex. ratio	A	−0.251	0.096	−0.095	0.535	−0.199	0.191
	B	−0.228	0.137	−0.097	0.532	−0.193	0.208
	C	−0.246	0.112	−0.097	0.536	−0.221	0.154
	D	−0.232	0.129	−0.098	0.527	−0.199	0.194

Trunk flex./ret. mean	A	−0.114	0.455	0.177	0.246	0.033	0.83
	B	−0.126	0.416	0.227	0.139	0.089	0.565
	C	−0.084	0.591	0.257	0.096	0.112	0.474
	D	−0.232	0.13	0.246	0.107	0.038	0.808

Rotation right mean	A	−0.242	0.109	0.146	0.337	0.137	0.369
	B	−0.168	0.277	0.103	0.506	0.153	0.322
	C	−0.203	0.192	0.275	0.075	0.205	0.187
	D	−0.141	0.362	0.233	0.127	0.201	0.191

Rotation sym.	A	−0.188	0.216	0.161	0.291	0.134	0.379
	B	−0.015	0.924	0.176	0.254	0.223	0.147
	C	−0.144	0.358	0.187	0.231	0.224	0.148
	D	−0.142	0.363	0.188	0.226	0.227	0.143

Rotation L mean	A	−0.194	0.202	0.152	0.319	0.12	0.432
	B	−0.161	0.297	0.078	0.616	0.21	0.171
	C	−0.187	0.229	0.171	0.272	0.236	0.127
	D	−0.212	0.171	0.213	0.171	0.201	0.195

Lat. flex. right mean	A	−0.203	0.181	0.261	0.083	0.193	0.204
	B	−0.044	0.776	0.261	0.087	0.362	0.016
	C	0.039	0.806	0.128	0.412	0.362	**0.017**
	D	−0.098	0.531	0.175	0.261	0.29	0.059

Lat. flex. sym.	A	−0.089	0.563	−0.033	0.831	−0.026	0.866
	B	−0.169	0.274	0.134	0.387	0.177	0.249
	C	−0.052	0.743	0.126	0.421	0.238	0.124
	D	−0.234	0.131	0.169	0.279	0.19	0.222

Lat. flex. left mean	A	−0.126	0.409	−0.031	0.841	−0.014	0.928
	B	−0.008	0.959	0.186	0.227	0.279	0.067
	C	−0.045	0.772	0.197	0.204	0.255	0.099
	D	−0.105	0.501	0.213	0.17	0.166	0.288
